# Supplementary material for: Top-down modulation of sensory processing and mismatch in the mouse posterior parietal cortex
Source: Nat Commun. 2025 May 7;16:4240. doi: 10.1038/s41467-025-58002-2 (PMC12059120; doi:10.1038/s41467-025-58002-2)
Supplement: Supplementary file 1 — Supplementary Information [file 41467_2025_58002_MOESM1_ESM.pdf]

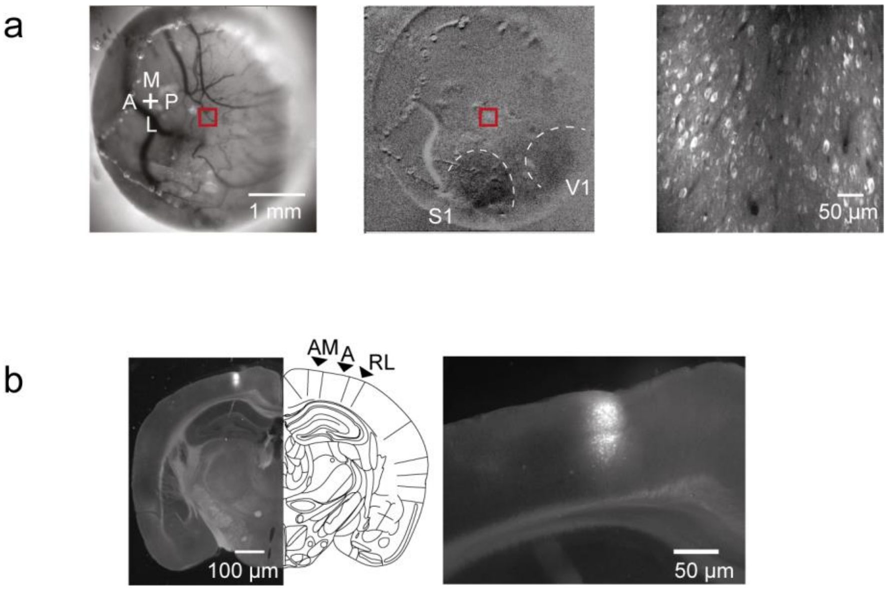

Supplementary Fig. 1. Locating area A of the PPC through intrinsic optical signal imaging (IOS). **a** Left, view of a chronically implanted cranial window over the PPC, with the imaging site outlined in red. Center, location of the γ and δ -whisker barrel in S1 and of V1, as determined with IOS seen through a 4-mm cranial window. Right, view of the imaged field of view (red box) of layer 2/3 neurons expressing the GECI RCaMP1.07, as acquired with the 2-photon microscope. **b** Left, transverse section of a brain slice with RCaMP1.07 expression in layers 2/3 of the PPC. Right, a close-up of the GECI expression site. The schematic drawings of the brain in panel **b** are used with permission of Elsevier from The Mouse Brain in Stereotaxic Coordinates, Paxinos, G., Franklin, K. B. J., 2 edition, page 30, Academic Press, 2001; permission conveyed through Copyright Clearance Center, Inc.

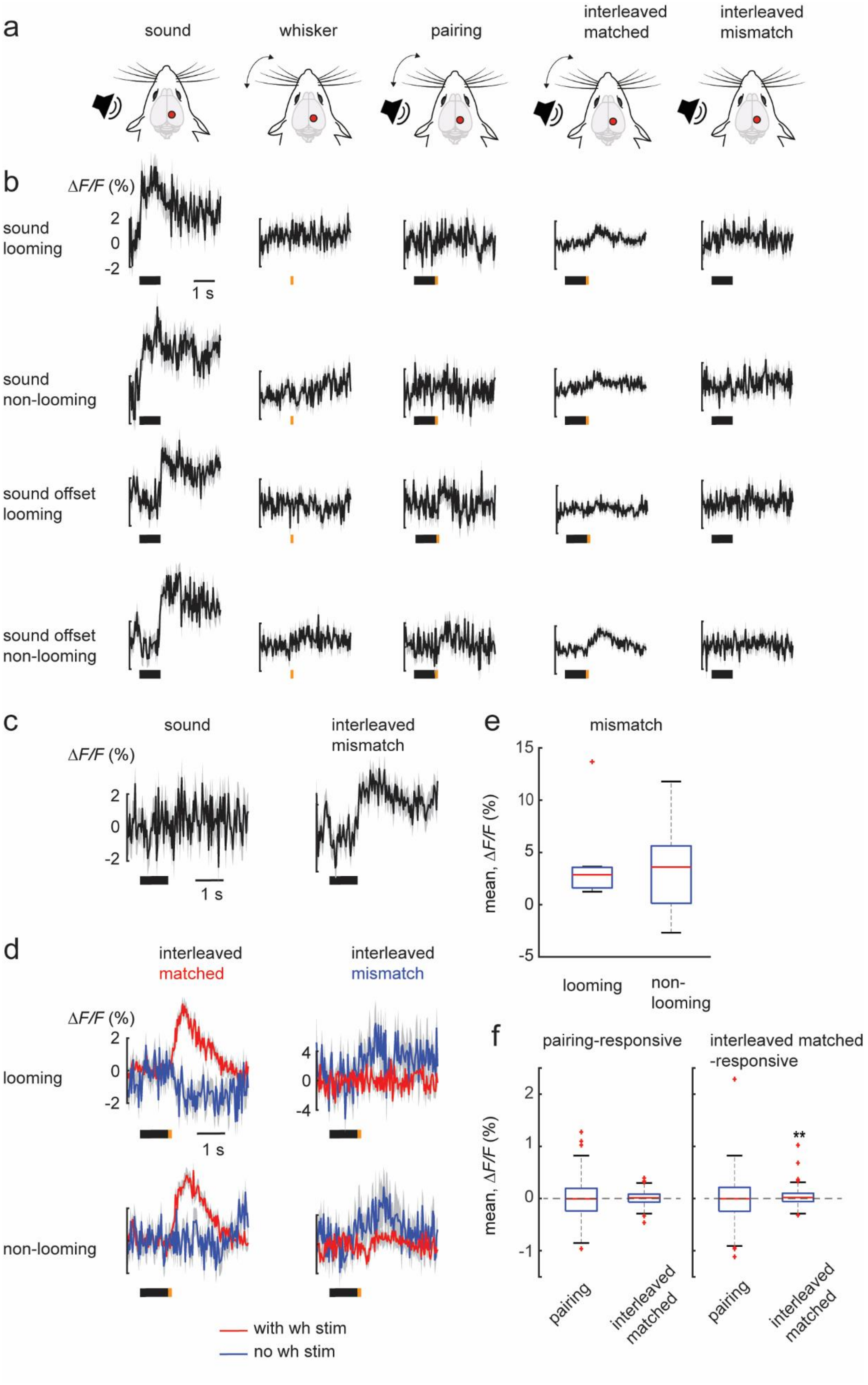

Supplementary Fig. 2. Creating a sensory association at the PPC.

a Schematic of the sequence of stimulus presentation (same as Fig. 1a). **b** Same as **Fig. 1d**, for looming sound (101 neurons), non-looming sound (103 neurons), looming sound offset-responsive (n = 98) and

non-looming sound offset-responsive (117 neurons) neurons. **c** Average population responses of mismatch-responsive neurons (from Fig. 1d) in their corresponding looming sound trials. Note the absence of a sound offset response in the sound trials for these neurons. **d** Population averages (± s.e.m.) of $\Delta F/F$ traces of interleaved matched and mismatch-responsive neurons when the whisker stimulus is associated with a looming and non-looming sound respectively. **e** Boxplots of average population responses of mismatch-responsive neurons, with a looming (9 neurons) and non-looming (11 neurons) sound. wh stim: whisker stimulus. **f** Boxplot from Fig. 1g with outliers. Data represented as mean ± s.e.m. The mouse illustration in panel **a** was adapted from an image created in the laboratory of Carl Petersen, EPFL.

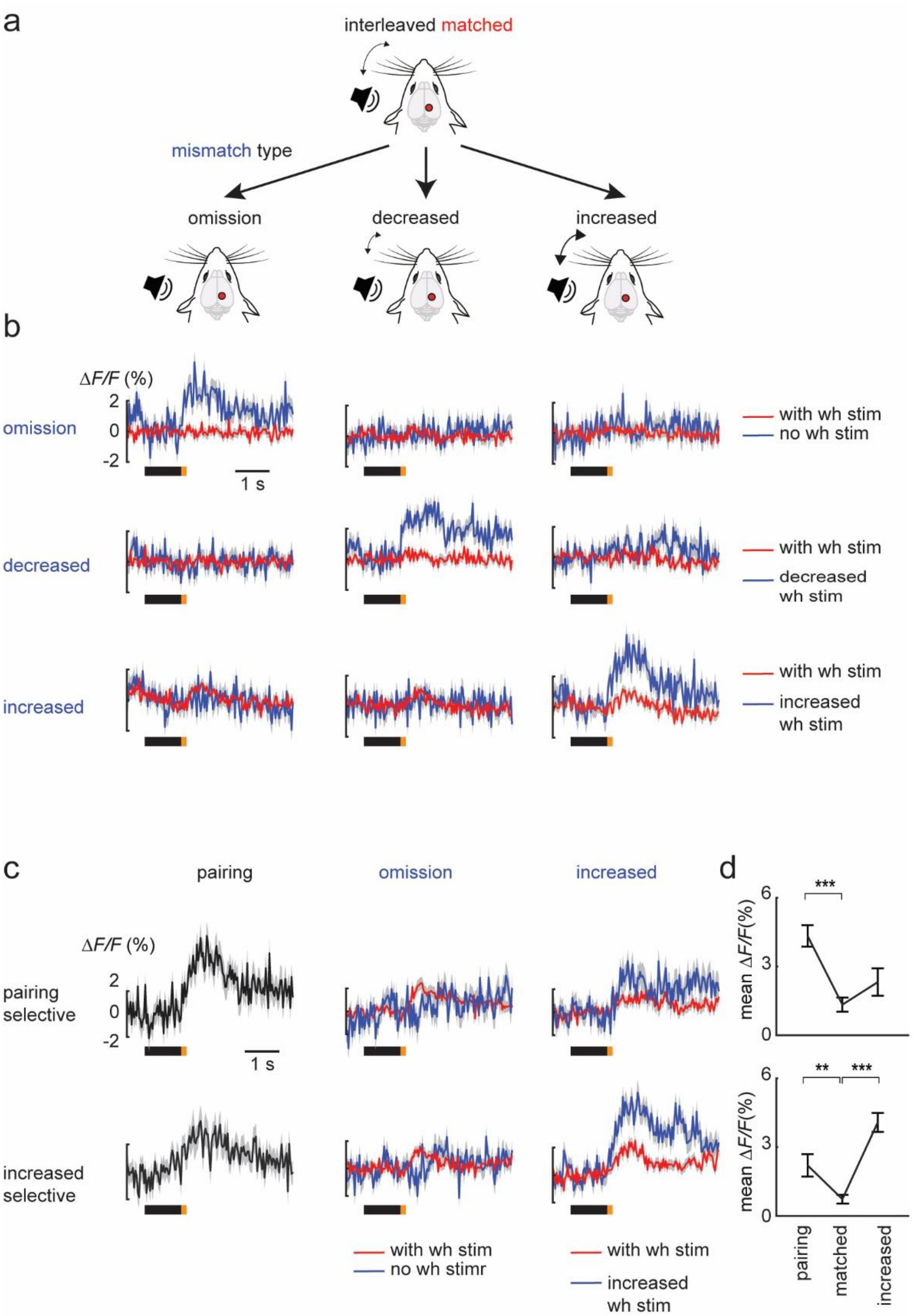

Supplementary Fig. 3. PPC neurons can report different types of mismatch.

a-**b** Same as **Fig. 3e-f**, with corresponding population average traces across the different mismatch trials for each mismatch selective population. **C** Population averages (± s.e.m.) of ΔF/F traces of pairing (65 neurons) and increased-responsive (mismatch) neurons (114 neurons), across the pairing, omission-mismatch and increased-mismatch sessions. **d** Average population responses of the neurons in **c** across the sessions. wh stim: whisker stimulus. Data represented as mean ± s.e.m. Statistical significance is indicated by ** for $p < 0.01$, and *** for $p < 0.001$, with two-sided Wilcoxon signed-rank

paired test. The mouse illustration in panel **a** was adapted from an image created in the laboratory of Carl Petersen, EPFL.

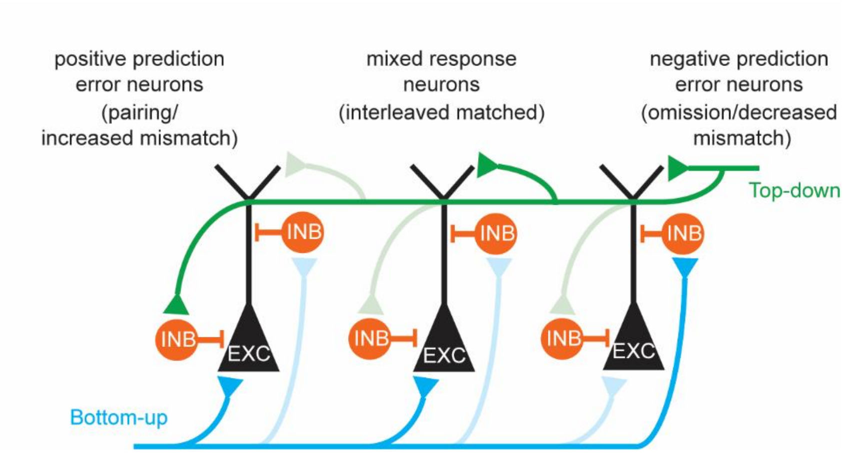

Supplementary Fig. 4. Microcircuit for predictive processing in the PPC.

We have adapted the proposed canonical microcircuit for predictive processing to include the respective session-responsive neurons that we have identified, in this simplified model restricted to layer 2/3 neurons. In this model, the positive prediction error neurons receive bottom-up input in the form of the whisker stimulus, which is balanced by top-down inhibition. The pairing-responsive neurons could contribute to part of this class of positive prediction error neurons. They are suppressed in transition from the pairing to interleaved session, as the prediction is formed. These pairing-responsive neurons can than report the increased-mismatch (Supplementary **Fig. 3c-d**), where the whisker stimulus is stronger than expected, compared to the interleaved matched trials (positive prediction error). The interleaved matched-responsive neurons increase their response to the whisker stimulus in transition from the pairing to the interleaved session, as they now receive more top-down feedback/prediction (matched with an increased pre-stimulus response in **Fig. 2h**). Lastly the negative prediction error neurons receive top-down feedback that is balanced by bottom-up inhibition. Both the omission and decreased intensity mismatch-responsive neurons could represent these negative prediction error neurons as they are recruited during the omission of the whisker stimulus and the presentation of a decreased whisker stimulus intensity respectively.

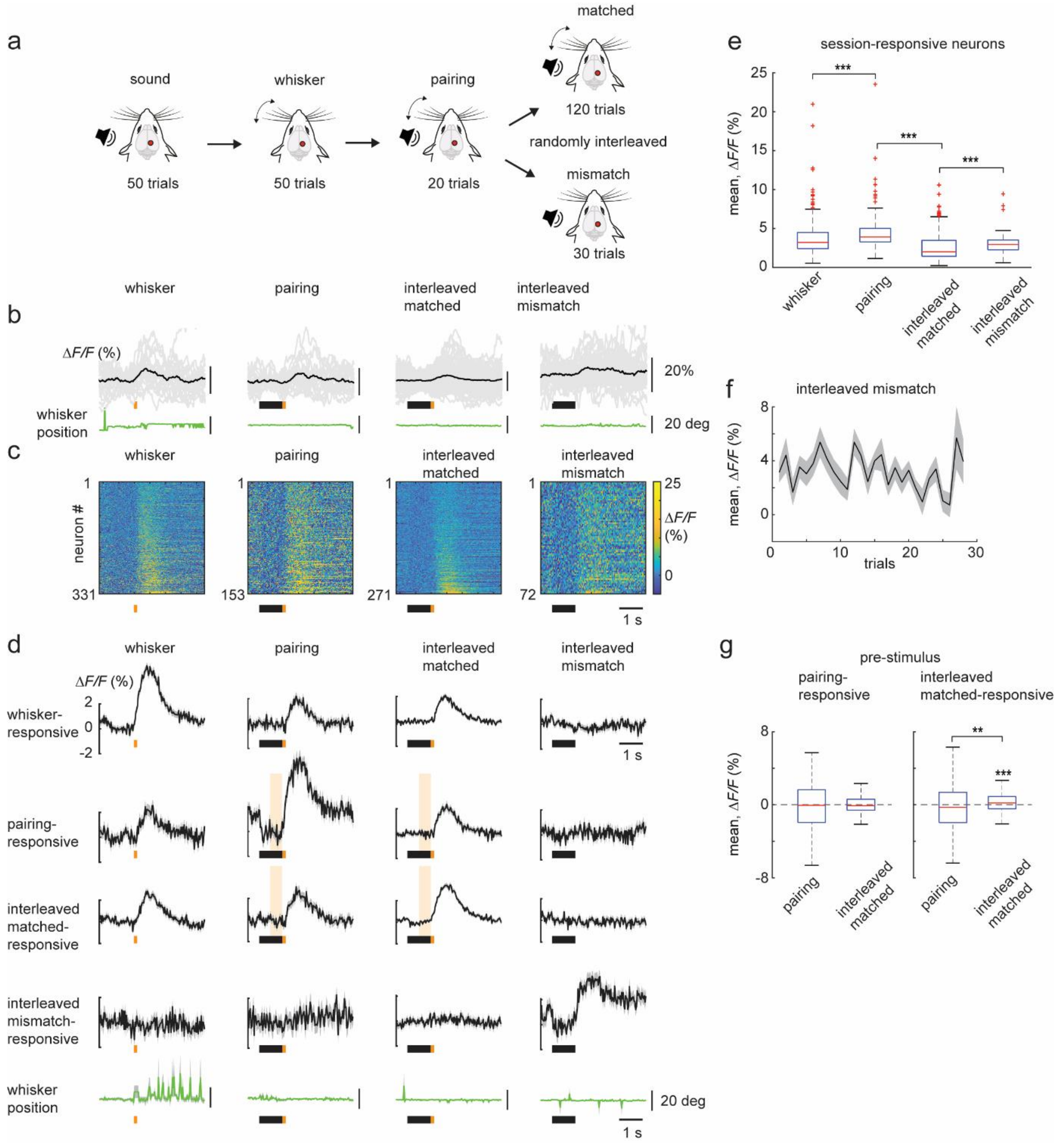

Supplementary Fig. 5 Creating a sensory association at the PPC. a Schematic of the sequence of stimulus presentation carried out in a single imaging session. All trials begin with a brief sound cue (not shown). Looming and non-looming sounds are randomly interleaved in the sound trials. The whisker stimulus (orange dot in **b**) delivered by a magnetic coil is presented alone in the whisker session. The looming sound (black bar in **b**) is paired with the whisker stimulus in pairing session. In the interleaved session, the matched trials (looming sound followed by whisker stimulus) are randomly interleaved with the mismatch trials where the whisker stimulus is omitted. **b** Example stimulus-responsive neurons classified as whisker-, pairing-, interleaved matched-, and interleaved mismatch-responsive neurons from layer 2/3 of the PPC, expressing RCaMP1.07. Average (black) and single trial (grey) ΔF/F calcium traces are shown, along with the corresponding average whisker position (green). **c** Heat maps representing the average stimulus responses of whisker- (331 neurons), pairing- (153 neurons), interleaved matched- (271 neurons) and interleaved mismatch-responsive neurons (72 neurons) from 6

wild-type mice. Mean pre-stimulus activity (1 s) was subtracted from the calcium transients and neurons were sorted to their mean ΔF/F response times in the stimulus window. **d** Population averages (± s.e.m.) of ΔF/F traces of the neurons in **c**, along with their corresponding population average for the other presented stimuli. The average whisker position from the above sessions is shown below in green. **e** Box plot of average population responses of whisker-, pairing-, matched- and mismatch-responsive neurons. **f** Average population responses of mismatch-responsive neurons over mismatch trials. **g** Boxplot of pre-whisker stimulus response of pairing- and interleaved matched-responsive neurons, during their pairing and interleaved-matched sessions. Pre-stimulus response corresponds to shaded area in **d**. Data are represented as mean ± s.e.m. in **f**. Statistical significance is indicated by ** for $p < 0.01$ and *** for $p < 0.001$, with two-sided Wilcoxon-Mann-Whitney test. The mouse illustration in panel **a** was adapted from an image created in the laboratory of Carl Petersen, EPFL.

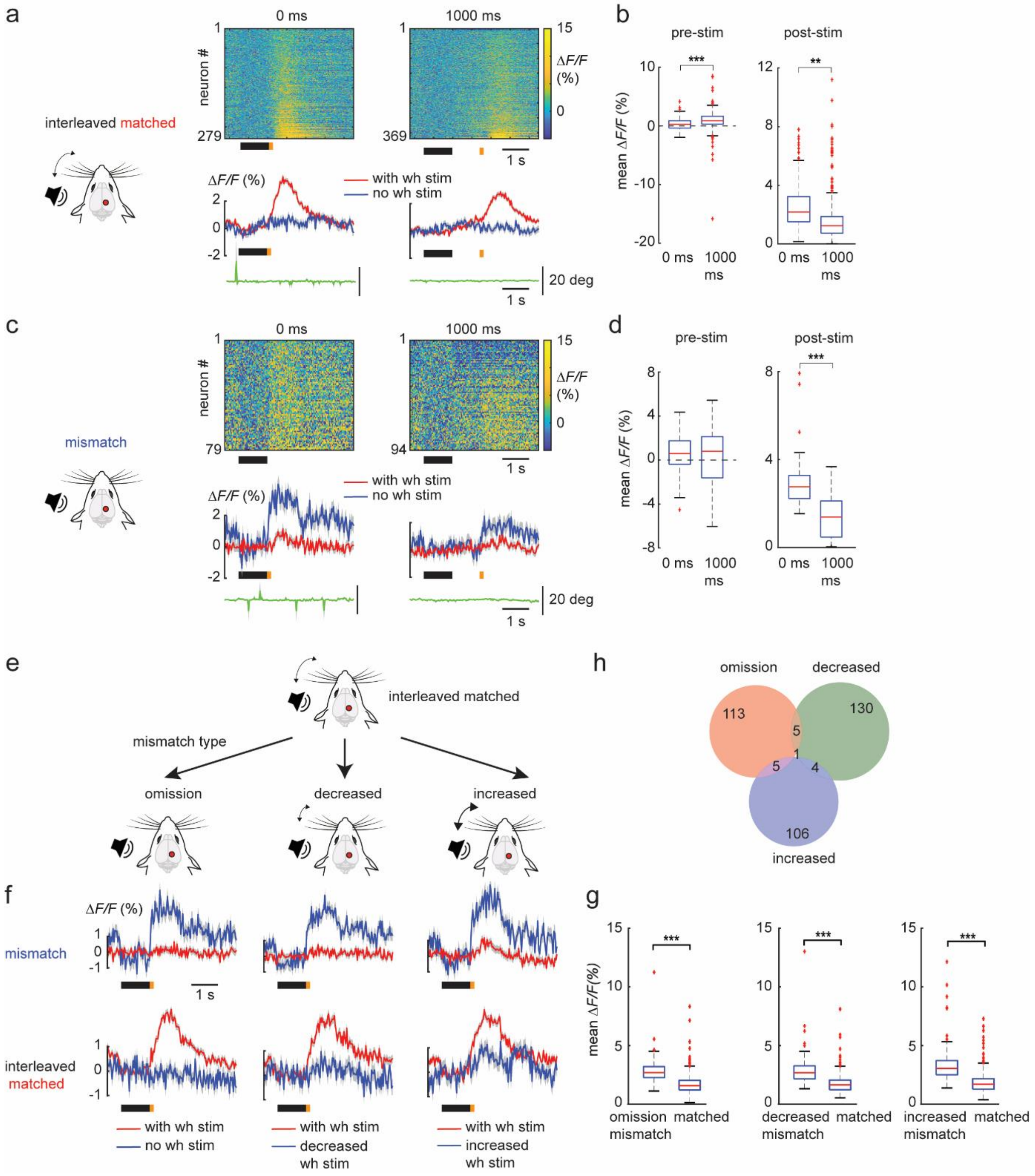

Supplementary Fig. 6 PPC can reliably update the mismatch response and report different types of mismatch. A Heat maps and corresponding population averages (± s.e.m.) of ΔF/F traces of interleaved matched-responsive neurons with no delay (0 ms) and a 1000 ms delay between the looming sound and whisker stimulus presentation (without delay, 279 neurons; with delay, 369 neurons; 6 mice; 6 FOVs). The average whisker position from the above sessions is shown below in green. **b** Box plot of average population pre-stimulus and post-stimulus responses of interleaved matched-responsive neurons, without and with delay. **c**–**d** same as **a**–**b**, but for mismatch-responsive neurons (without delay, 79 neurons; with delay, 94 neurons). **e** Schematic of the presentation of different mismatch trial types in the form of whisker stimulus omission, decreased and increased whisker stimulus intensity. **f** Population averages (± s.e.m.) of ΔF/F traces of interleaved mismatch-responsive neurons and matched-responsive neurons, for the interleaved sessions with omission (124 mismatch neurons, 307 matched

neurons), decreased (140 mismatch neurons, 217 matched neurons) and increased (116 mismatch neurons, 316 matched neurons) whisker stimulus mismatches, with their corresponding matched (red) and mismatch (blue) trial averages. **g** Box plot of average population responses of mismatch-responsive and matched-responsive neurons, for the interleaved sessions with omission, decreased and increased whisker stimulus mismatches. **h** Venn diagram of the different mismatch-responsive neurons in **f**, their respective overlaps between mismatch sessions. wh stim: whisker stimulus. Data are represented as mean ± s.e.m. Statistical significance is indicated by ** for $p < 0.01$, and *** for $p < 0.001$, with two-sided Wilcoxon-Mann-Whitney test. The mouse illustrations in panels **a**, **c** and **e** were adapted from an image created in the laboratory of Carl Petersen, EPFL.

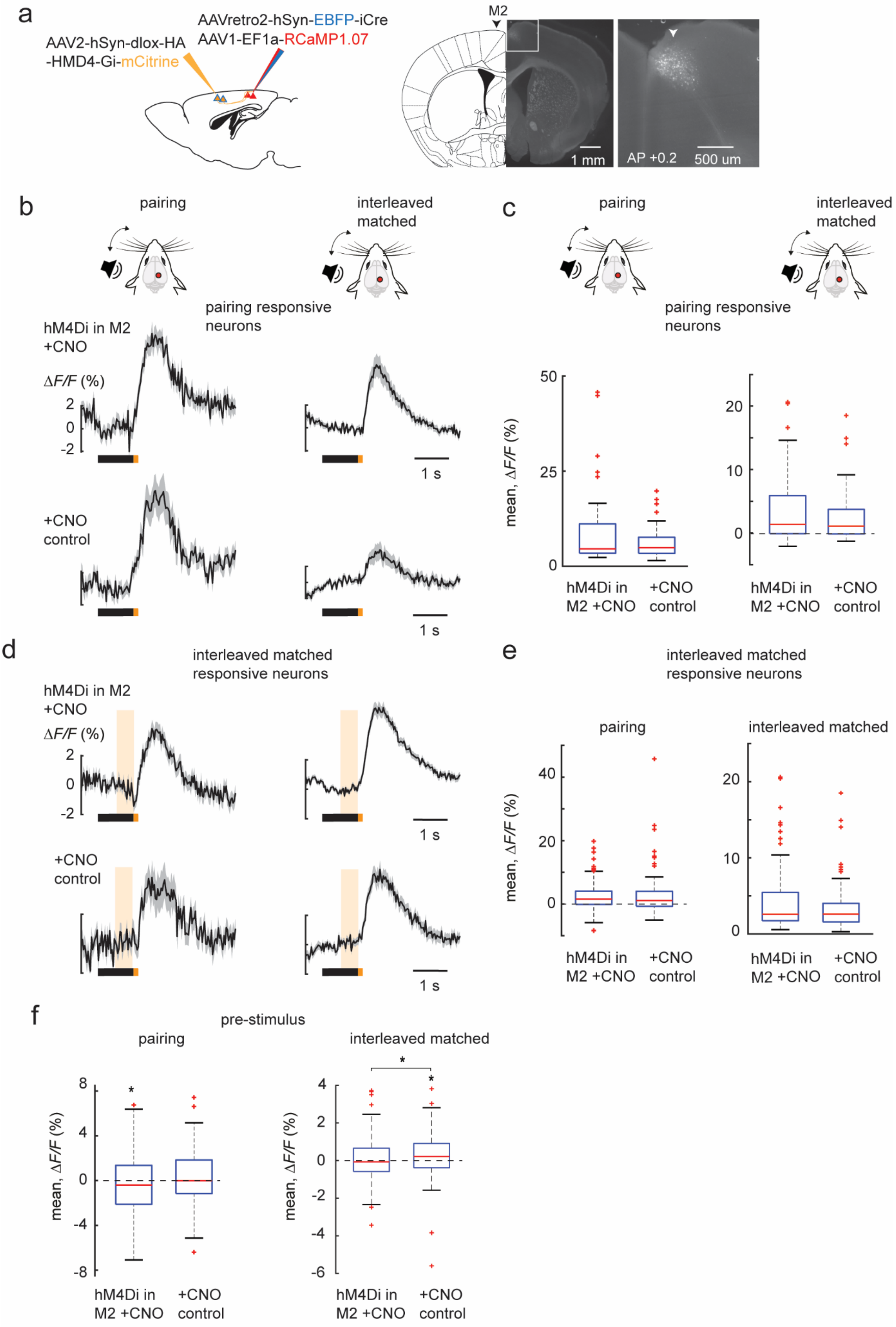
a
AAV2-hSyn-dlox-HA
-HMD4-Gi-mCitrine
AAVretro2-hSyn-EBFP-iCre
AAV1-EF1a-RCaMP1.07
M2
1 mm
AP +0.2
500 um
b
pairing
interleaved
matched
pairing responsive
neurons
hM4Di in M2
+CNO
ΔF/F (%)
2
0
-2
1 s
+CNO
control
1 s
c
pairing
interleaved
matched
pairing responsive
neurons
50
25
0
mean, ΔF/F (%)
20
10
0
hM4Di in
M2 +CNO
+CNO
control
hM4Di in
M2 +CNO
+CNO
control
d
interleaved matched
responsive neurons
hM4Di in M2
+CNO
ΔF/F (%)
2
0
-2
1 s
+CNO
control
1 s
e
interleaved matched
responsive neurons
pairing
interleaved matched
40
20
0
mean, ΔF/F (%)
20
10
0
hM4Di in
M2 +CNO
+CNO
control
hM4Di in
M2 +CNO
+CNO
control
f
pre-stimulus
pairing
interleaved matched
8
4
0
-4
-8
mean, ΔF/F (%)
*
4
2
0
-2
-4
-6
mean, ΔF/F (%)
*
*
hM4Di in
M2 +CNO
+CNO
control
hM4Di in
M2 +CNO
+CNO
control

Supplementary Fig. 7 M2 modulates sensory processing in the PPC. a Injection scheme to express the inhibitory DREADD hM4Di in M2 neurons projecting to the PPC (sagittal view), along with a coronal section showing their expression in M2 neurons as represented by mCitrine fluorescence. **b** Population averages (± s.e.m.) of ΔF/F traces of pairing-responsive neurons, in the pairing and interleaved matched sessions, for the hM4Di+CNO (3 mice, 6 FOVs, 80 neurons) and +CNO control experiments (4 mice, 6 FOVs, 49 neurons). **c** Box plot of post-whisker stimulus response difference between interleaved matched and pairing sessions in the pairing-responsive neurons in **b**. **d** Population averages (± s.e.m.) of ΔF/F traces of interleaved matched-responsive neurons, in the pairing and interleaved matched sessions, for the hM4Di+CNO (195 neurons) and +CNO control experiments (90 neurons). **e** Box plot of post-whisker stimulus response difference between interleaved matched and pairing sessions in the interleaved matched-responsive neurons in **d**. **f** Box plot of average population pre-stimulus responses of the interleaved matched-responsive neurons in **d**, for their pairing and interleaved matched sessions. Data are represented as mean ± s.e.m. Statistical significance is indicated by * for $p < 0.05$ with two-sided Wilcoxon-Mann-Whitney test. The schematic drawings of the brain in panel **a** are used with permission of Elsevier from The Mouse Brain in Stereotaxic Coordinates, Paxinos, G., Franklin, K. B. J., 2 edition, page 47, Academic Press, 2001; permission conveyed through Copyright Clearance Center, Inc. The mouse illustration in panel **b** was adapted from an image created in the laboratory of Carl Petersen, EPFL.

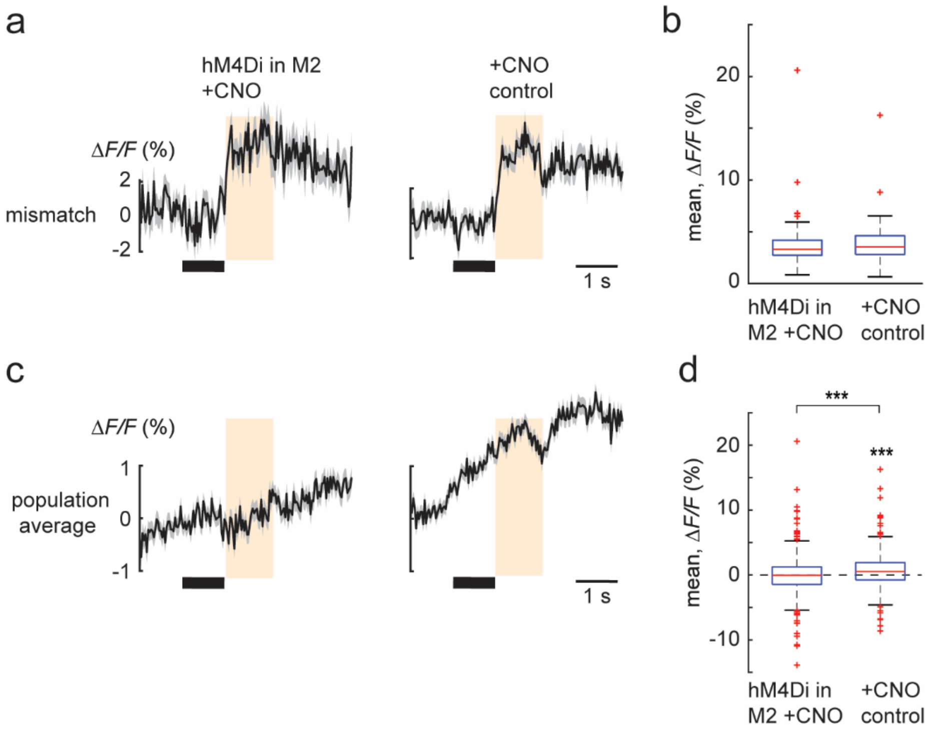

Supplementary Fig. 8 M2 contributes to the top-down prediction to the PPC. a Population averages (± s.e.m.) of ΔF/F traces of interleaved mismatch-responsive neurons in layer 2/3 neurons in the PPC with M2 suppression using hM4Di+CNO (42 neurons) and with +CNO control experiments (78 neurons) (same mice as in Fig. 4). **b** Box plot of average population responses of the interleaved mismatch-responsive neurons in **a**. **c** Population averages (± s.e.m.) of ΔF/F traces of all neurons during the mismatch trials in hM4Di+CNO (n = 1014 neurons) and +CNO control experiments (n = 882 neurons). **d** Box plot of average population responses of the neurons in **c** during the mismatch trials. Data are represented as mean ± s.e.m. Statistical significance is indicated by *** for $p < 0.001$, with two-sided Wilcoxon-Mann-Whitney test.
