# Supplementary material for: Top-down modulation of sensory processing and mismatch in the mouse posterior parietal cortex
Source: Nat Commun. 2025 May 7;16:4240. doi: 10.1038/s41467-025-58002-2 (PMC12059120; doi:10.1038/s41467-025-58002-2)
Supplement: Supplementary file 2 — Reporting Summary [file 41467_2025_58002_MOESM2_ESM.pdf]

nature portfolio

Corresponding author(s): Shankar Sachidhanandam

Last updated by author(s): Jan 8, 2025

Reporting Summary

Nature Portfolio wishes to improve the reproducibility of the work that we publish. This form provides structure for consistency and transparency in reporting. For further information on Nature Portfolio policies, see our Editorial Policies and the Editorial Policy Checklist.

Statistics

For all statistical analyses, confirm that the following items are present in the figure legend, table legend, main text, or Methods section.

n/a	Confirmed	
☐	☒	The exact sample size (*n*) for each experimental group/condition, given as a discrete number and unit of measurement
☐	☒	A statement on whether measurements were taken from distinct samples or whether the same sample was measured repeatedly
☐	☒	The statistical test(s) used AND whether they are one- or two-sided<br>*Only common tests should be described solely by name; describe more complex techniques in the Methods section.*
☐	☒	A description of all covariates tested
☐	☒	A description of any assumptions or corrections, such as tests of normality and adjustment for multiple comparisons
☐	☒	A full description of the statistical parameters including central tendency (e.g. means) or other basic estimates (e.g. regression coefficient) AND variation (e.g. standard deviation) or associated estimates of uncertainty (e.g. confidence intervals)
☐	☒	For null hypothesis testing, the test statistic (e.g. *F*, *t*, *r*) with confidence intervals, effect sizes, degrees of freedom and *P* value noted<br>*Give P values as exact values whenever suitable.*
☒	☐	For Bayesian analysis, information on the choice of priors and Markov chain Monte Carlo settings
☒	☐	For hierarchical and complex designs, identification of the appropriate level for tests and full reporting of outcomes
☒	☐	Estimates of effect sizes (e.g. Cohen's *d*, Pearson's *r*), indicating how they were calculated

Our web collection on statistics for biologists contains articles on many of the points above.

Software and code

Policy information about availability of computer code

Data collection	Neuronal imaging data was acquired with ScanImage 2019 running on Matlab2016a. Whisker position tracking was done with PixyMon.
Data analysis	Calcium signals were extracted using the Python based CaImAn analysis pipeline. Subsequent data analysis was performed using custom routines in Matlab2022b. Custom scripts are available on Zenodo (https://doi.org/10.5281/zenodo.14192397)

For manuscripts utilizing custom algorithms or software that are central to the research but not yet described in published literature, software must be made available to editors and reviewers. We strongly encourage code deposition in a community repository (e.g. GitHub). See the Nature Portfolio guidelines for submitting code & software for further information.

Data

Policy information about availability of data

All manuscripts must include a data availability statement. This statement should provide the following information, where applicable:

- Accession codes, unique identifiers, or web links for publicly available datasets
- A description of any restrictions on data availability
- For clinical datasets or third party data, please ensure that the statement adheres to our policy

The data generated from this study to reach the conclusions are available on Zenodo (https://doi.org/10.5281/zenodo.14192397)

Research involving human participants, their data, or biological material

Policy information about studies with human participants or human data. See also policy information about sex, gender (identity/presentation), and sexual orientation and race, ethnicity and racism.

Reporting on sex and gender	This study did not involve human participants.
Reporting on race, ethnicity, or other socially relevant groupings	This study did not involve human participants.
Population characteristics	This study did not involve human participants.
Recruitment	This study did not involve human participants.
Ethics oversight	This study did not involve human participants.

Note that full information on the approval of the study protocol must also be provided in the manuscript.

Field-specific reporting

Please select the one below that is the best fit for your research. If you are not sure, read the appropriate sections before making your selection.

☒ Life sciences ☐ Behavioural & social sciences ☐ Ecological, evolutionary & environmental sciences

For a reference copy of the document with all sections, see nature.com/documents/nr-reporting-summary-flat.pdf

Life sciences study design

All studies must disclose on these points even when the disclosure is negative.

Sample size	No statistical methods were used to predetermine the sample size. The sample sizes that we used in the tests gave statistical powers higher than 0.9.
Data exclusions	No data were excluded from the analysis.
Replication	We were able to replicate our main findings using two different calcium indicators, in wild-type and transgenic Thy-1GCaMP6f mice.
Randomization	No randomization was done as the mice were not separated into control and experimental groups.
Blinding	No blinding was done during the acquisition of the data. The experiments were performed in a sequential manner that was consistent across the mice.

Reporting for specific materials, systems and methods

We require information from authors about some types of materials, experimental systems and methods used in many studies. Here, indicate whether each material, system or method listed is relevant to your study. If you are not sure if a list item applies to your research, read the appropriate section before selecting a response.

Materials & experimental systems

n/a	Involved in the study	
☒	☐	Antibodies
☒	☐	Eukaryotic cell lines
☒	☐	Palaeontology and archaeology
☐	☒	Animals and other organisms
☒	☐	Clinical data
☒	☐	Dual use research of concern
☒	☐	Plants

Methods

n/a	Involved in the study	
☒	☐	ChIP-seq
☒	☐	Flow cytometry
☒	☐	MRI-based neuroimaging

Animals and other research organisms

Policy information about studies involving animals; ARRIVE guidelines recommended for reporting animal research, and Sex and Gender in Research

Laboratory animals	Mice: C57/BL6, males and females, 8 to 15 weeks old
Wild animals	No wild animals were used in this study
Reporting on sex	Male and female mice were used in this study. The study design did not take into account the sex of the animals.
Field-collected samples	No samples were collected from the field in this study.
Ethics oversight	All experimental procedures followed the guidelines of the Veterinary Office of Switzerland and were approved by the Cantonal Veterinary Office in Bern.

Note that full information on the approval of the study protocol must also be provided in the manuscript.
